# Leisure-Time Physical Activity and Cancer Mortality Among Cancer Survivors

**DOI:** 10.1001/jamanetworkopen.2025.56971

**Published:** 2026-02-17

**Authors:** Erika Rees-Punia, Lauren R. Teras, Christina C. Newton, Steven C. Moore, I-Min Lee, Lauren Bates-Fraser, Kathryn E. Chiang, Den E. Bloodworth, A. Heather Eliassen, Lorelei Mucci, Brigid M. Lynch, Meir Stampfer, Mingyang Song, Kristen D. Brantley, Konrad H. Stopsack, Charles E. Matthews, Alpa V. Patel

**Affiliations:** 1 Department of Population Science, American Cancer Society, Atlanta, Georgia; 2 Division of Cancer Epidemiology and Genetics, National Cancer Institute, Rockville, Maryland; 3 Brigham and Women’s Hospital, Harvard Medical School, Boston, Massachusetts; 4 Department of Epidemiology, Harvard T.H. Chan School of Public Health, Boston, Massachusetts; 5 Department of Nutrition, Harvard T.H. Chan School of Public Health, Boston, Massachusetts; 6 Channing Division of Network Medicine, Department of Medicine, Brigham and Women’s Hospital and Harvard Medical School, Boston, Massachusetts; 7 Discovery Science, American Cancer Society, Atlanta, Georgia; 8 Cancer Epidemiology Division, Cancer Council Victoria, Melbourne, Victoria, Australia; 9 Centre for Epidemiology and Biostatistics, Melbourne School of Population and Global Health, University of Melbourne, Melbourne, Victoria, Australia; 10 Division of Gastroenterology, Harvard Medical School, Boston, Massachusetts; 11 Clinical and Translational Epidemiology Unit, Massachusetts General Hospital, Boston; 12 Department of Medical Oncology, Dana-Farber Cancer Institute, Boston, Massachusetts; 13 Department of Epidemiologic Methods and Etiologic Research, Leibniz Institute for Prevention Research and Epidemiology–BIPS and Faculty of Human and Health Sciences, University of Bremen, Bremen, Germany

## Abstract

**Importance:**

There is insufficient evidence to determine whether physical activity lengthens survival among people with a history of cancers less commonly studied for such benefit.

**Objective:**

To examine the associations between physical activity assessed after a cancer diagnosis with cancer mortality and, secondarily, changes in physical activity before vs after diagnosis with cancer mortality among people previously diagnosed with 1 of 7 cancers.

**Design, Setting, and Participants:**

This study used a pooled dataset of 6 cohorts (Cancer Prevention Study-II Nutrition Cohort, Health Professionals Follow-Up Study, National Institutes of Health–AARP Diet and Health Study, Nurses’ Health Study, Nurses’ Health Study II, and Women’s Health Study). Participants were survivors of bladder, endometrial, kidney, lung, oral cavity, ovarian, or rectal cancer who had completed surveys and had repeated measures of leisure-time physical activity. Baseline data were collected from 1976 through 1997. The mean (SD) follow-up was 10.9 (7.0) years. Data were analyzed from June 2023 to March 2024.

**Exposures:**

Leisure-time moderate to vigorous physical activity (MVPA) before and after cancer diagnosis.

**Main Outcomes and Measures:**

Association of MVPA in categories of metabolic equivalents of task hours per week (MET-h/wk) measured before and a mean (SD) of 2.8 (1.5) years after cancer diagnosis with cancer mortality.

**Results:**

This pooled analysis included 17 141 cancer survivors (mean [SD] age, 67 [8] years; 60% female). Engagement in low amounts of MVPA (>0 to <7.5 vs 0 MET-h/wk) was associated with lower risk of cancer mortality among survivors who had been diagnosed with bladder (hazard ratio [HR], 0.67 [95% CI, 0.50-0.91]), endometrial (HR, 0.62 [95% CI, 0.45-0.87]), and lung cancer (HR, 0.56 [95% CI, 0.43-0.75]). Doubling the recommended MVPA guideline or more (eg, >15 vs 0 MET-h/wk) was associated with lower risk of cancer mortality among oral (HR, 0.39 [95% CI, 0.15-0.99] for >22.5 to 30.0 MET-h/wk) and rectal (HR, 0.57 [95% CI, 0.33-0.97] for >15.0 to 22.5 MET-h/wk) cancer survivors. Point estimates were less than 1 for cancer mortality among kidney cancer survivors (HR, 0.51 [95% CI, 0.22-1.18] for >15.0 to 22.5 MET-h/wk), although the confidence interval included the null. Compared with survivors who did not meet the MVPA guidelines before or after diagnosis, lung (HR, 0.58 [95% CI, 0.47-0.71]) and rectal (HR, 0.51 [95% CI, 0.32-0.83]) cancer survivors who met guidelines after diagnosis had a lower risk of cancer mortality, even if they were inactive before their diagnosis.

**Conclusions and Relevance:**

In this analysis of 6 pooled cohorts, higher levels of MVPA after a cancer diagnosis were associated with lower risk of cancer mortality among people previously diagnosed with 1 of 7 cancers not commonly studied for their association with MVPA. Findings suggest that it is important for health care professionals to promote physical activity for longevity and overall health among people living with and beyond cancer.

## Key Points

**Question:**

Is engagement in physical activity after a cancer diagnosis associated with longer survival among individuals with a history of bladder, endometrial, kidney, lung, oral, ovarian, or rectal cancer?

**Findings:**

This pooled analysis of 6 cohort studies involving 17 141 participants found that higher levels of moderate to vigorous physical activity after diagnosis were associated with lower risk of cancer mortality among survivors of bladder, endometrial, lung, and ovarian cancers. Lung and rectal cancer survivors who were inactive before diagnosis but became active after diagnosis had lower risk of cancer mortality.

**Meaning:**

Findings suggest that physical activity may benefit survivors of cancer, even if they were inactive prior to diagnosis.

## Teaser

This pooled analysis of 6 cohort studies evaluates whether participating in moderate to vigorous physical activity after a cancer diagnosis is associated with longer survival among individuals with a history of bladder, endometrial, kidney, lung, oral, ovarian, or rectal cancer.

## Introduction

The role of physical activity (PA) in mitigating cancer risk is well recognized,^1,2,3,4,5^ and the understanding of this association has led to clear PA recommendations for cancer prevention.^6^ Similarly, people with a history of cancer are recommended to accumulate 150 to 300 minutes of moderate intensity or 75 to 150 minutes of vigorous intensity aerobic PA per week (eg, 7.5-15.0 metabolic equivalent of task hours per week [MET-h/wk]).^7^ However, these recommendations are largely based on research of mortality outcomes within breast,^8,9,10,11,12^ prostate,^13,14,15^ or colon^16,17,18^ cancer survivors. Indeed, at the time of the United States Physical Activity Guidelines Report on physical activity and cancer (2019),^3^ systematic reviews, meta-analyses, and pooled analyses on the association between physical activity and mortality among cancer survivors were available only for survivors of these 3 cancer types. Thus, there is insufficient evidence to support appropriate PA recommendations for cancer survival among people with a history of cancers whose associations with PA and mortality have been less commonly studied. This is particularly problematic because roughly 50% of cancer cases are types other than breast, colon, and prostate. In fact, survivors of bladder, endometrial, kidney, lung, oral, ovarian, and rectal cancers together will account for over 30% of the 2 million new cancer cases in the US in 2025.^19,20^

The few recent studies of PA and mortality that include people with a history of cancers beyond breast, colon, and prostate^21,22^ provide promising evidence but are limited by a modest number of survivors of specific cancer types (eg, <12 000 survivors of all cancer types combined, including breast, colon, and prostate). These studies either combine survivors of less common cancers into a single category^21^ or explore cancer type–specific associations of PA and mortality in broad categories of PA, such as meeting vs not meeting PA guidelines.^22^ Highlighting gaps in earlier work, a 2019 meta-analysis of the association between postdiagnostic PA and cancer mortality only included 1 study among kidney cancer survivors, and no studies among bladder, ovarian, endometrial, lung, or oral cancer survivors.^23^ Taken together, it is clear that more research regarding the benefits of PA is needed among survivors of cancers other than breast, colon, and prostate. The aims of the present study were to examine the associations between PA assessed after a cancer diagnosis and before vs after diagnosis changes in PA with cancer mortality using a pooled dataset from bladder, endometrial, kidney, lung, oral, ovarian, or rectal cancer survivors.

## Methods

### Study Population

Cohorts participating in the National Cancer Institute Cohort Consortium Physical Activity and Cancer work group were considered for inclusion in this pooled analysis. Data from 6 cohorts that had repeated measures of leisure-time PA, confounding variables that were previously harmonized,^1,2^ and clinical data (including cancer treatment and stage) were pooled for the current analyses: the Cancer Prevention Study-II Nutrition Cohort (CPS-II NC),^24^ Health Professionals Follow-Up Study (HPFS),^25^ National Institutes of Health–AARP Diet and Health Study (NIH-AARP), Nurses’ Health Study (NHS),^26^ Nurses’ Health Study II (NHSII), and Women’s Health Study (WHS) (eTable 1 in Supplement 1).^27^ This study was conducted in accordance with the Strengthening the Reporting of Observational Studies in Epidemiology (STROBE) reporting guideline, and each cohort received ethical approval from its respective institutional review boards. The CPS-II NC protocol was approved by the institutional review boards of Emory University and of participating registries as required. The HPFS, NHS, WHS, and NHSII were conducted in accordance with the Declaration of Helsinki^28^ and approved by the institutional review board of Brigham and Women’s Hospital and Harvard T.H. Chan School of Public Health. The NIH-AARP study was approved by the Special Studies Institutional Review Board of the US National Cancer Institute, and all participants provided informed consent by completing and returning the baseline questionnaire. All participants provided written or oral informed consent. Baseline data were collected from 1976 through 1997.

Cancer site, stage, timing, and first-course treatment (chemotherapy and radiation therapy) were identified or confirmed by linking respective cohorts to cancer registries (CPS-II NC, NIH-AARP) or through medical record verification (CPS-II NC, NHS, NHSII, HPFS, and WHS). Given the focus on cancers less commonly studied for the association of PA with survival, survivors of cancers with at least 25 cancer deaths per cohort other than breast, colon, or prostate were included: bladder, endometrial (CPS-II NC, NIH-AARP, NHS, NHSII, and WHS only), kidney, lung, oral cavity, ovarian (CPS-II NC, NIH-AARP, NHS, NHSII, and WHS only), or rectal cancer (eTables 2-4 in Supplement 1).

### Physical Activity Exposures

All 6 cohorts assessed PA every 2 to 8 years over mean (SD) of 10.9 (7.0) years of follow-up. Estimated leisure-time moderate to vigorous PA (MVPA) from each cohort’s baseline survey (eTable 1 in Supplement 1) was used as the prediagnostic MVPA measure, and estimates from the first survey completed at least 1 year following the date of cancer diagnosis represented postdiagnostic MVPA. Assessments from this time frame were chosen to avoid estimates from surveys that may have been completed while undergoing active treatment, as treatment could impact the ability to achieve normal activity levels. Participants completed their MVPA survey a mean (SD) of 2.8 (1.5) years after their cancer diagnosis. Secondary and supplemental analyses also include a prediagnostic measure of MVPA, which was assessed on each cohort’s baseline survey (eTable 1 in Supplement 1).

MVPA surveys asked participants to report the average or approximate time spent per week on different recreational physical activities within the last year. Leisure-time activities classified as moderate (3.0-5.9 METs) or vigorous (≥6.0 METs) intensity were used to calculate the volume of MVPA in metabolic equivalent task hours per week (MET-h/wk). MVPA volume was classified according to current PA recommendations^7,29^ in the primary analyses: none (referent), more than 0 to less than 7.5 MET-h/wk (less than guideline or inactive), 7.5 to less than 15.0 MET-h/wk (meeting guideline), 15.0 to less than 22.5 MET-h/wk (doubling guideline), 22.5 to less than 30.0 MET-h/wk (tripling guideline), and ≥30.0 MET-h/wk (more than tripling guideline). In secondary analyses of prediagnosis to postdiagnosis change in MVPA, categories were combined to align with adherence to guidelines at each time point (ie, did not meet guideline prediagnosis or did not meet guideline postdiagnosis [referent], met guideline prediagnosis or did not meet guideline postdiagnosis).

### Mortality Outcomes

Vital status and cause of death were collected by individual cohorts via medical record review, death certificate, linkage to the US National Death Index, or reports from next of kin. The outcome was mortality with the primary cause of death being any type of cancer.

### Statistical Analysis

Cohort- and cancer site–specific hazard ratios (HRs) and 95% CIs for leisure-time MVPA and cancer mortality were estimated using Cox proportional hazards models and then pooled using DerSimonian and Laird random-effects models, consistent with 2-stage pooling methods.^30,31^ Delayed-entry Cox models, in which the time axis was time since diagnosis and follow-up time was calculated from postdiagnostic questionnaire return to death or the end of cohort follow-up, were used to account for differences in time between cancer diagnosis and postdiagnostic survey completion. A stratified Cox procedure was used to adjust for age within 1-year strata.

Model covariates were harmonized across cohorts and included self-reported sex, race and ethnicity (categorized by the research team as non-Latino White, all other races and ethnicities), smoking (never, former, current, and missing), and alcohol use (current user, nonuser, and missing) abstracted from the same survey as the MVPA exposure. Race and ethnicity were assessed on the baseline survey of each cohort for use as appropriate (eg, confounding variables, strata) in future analyses. Models also included stage at diagnosis (Surveillance, Epidemiology, and End Results General Summary Stage collapsed into in situ, localized, regional, distant, and missing [collapsed into distant, not distant, and missing for lung, oral, and bladder cancer survivors]; harmonized under the guidance of a certified oncology data specialist, D.E.B.) and first-course cancer treatment (chemotherapy: yes, no, or missing; and radiation: yes, no, or missing). Participants missing both cancer stage and treatment were excluded from analyses (n = 624). Prior work using this harmonized dataset compared results from models adjusting for cancer stage only, treatment only, and both stage and treatment, and there were only minor differences in model results.^32^ As cancer stage and treatment are potential mediators on a causal pathway from prediagnostic MVPA to survival, these variables were not included in supplemental models in which the main exposure was measured prior to diagnosis.^33^ Finally, models with or without adjustment for prediagnostic body mass index (BMI, calculated as weight in kilograms divided by height in meters squared) were compared, as BMI may be both a confounder and mediator of the association between MVPA and survival.

Because individuals with overt illness and impending death may reduce their activity levels, we conducted a sensitivity analysis excluding participants who died within 2 years of follow-up. Analyses restricted to participants diagnosed with in situ, local, or regional stage cancer were also conducted. Additionally, to reduce concerns around the association of residual confounding with smoking, analyses were repeated restricted to never smokers, although this analysis was only possible among bladder and endometrial cancer survivors because of the small number of cancer deaths among never smokers diagnosed with the other cancer types. The proportional hazards assumption was tested by assessing the interaction term between postdiagnostic MVPA and follow-up time within each cohort (eTable 5 in Supplement 1). All statistical analyses were conducted from June 2023 to March 2024 using SAS, version 9.4 (SAS Institute Inc). Statistical significance was defined as a 95% CI excluding 1.0.

## Results

The pooled analysis included 17 141 bladder, endometrial, kidney, lung, oral cavity, ovarian, or rectal cancer survivors with a postdiagnostic measure of MVPA (Table 1). Participants were a mean (SD) age of 67 (8) years at diagnosis. Overall, 49% of survivors were diagnosed with local or regional stage disease, and survivors of bladder (24%), endometrial (22%), and lung (18%) cancers accounted for over half of the cohort. Overall, 60% of participants in the pooled cohort were female (40% male). During a mean (SD) of 10.9 (7.0) years of follow-up, 4872 participants died of cancer.

**Table 1.** Cancer Survivor Characteristics by Postdiagnostic Leisure-Time Physical Activity

Characteristic	Survivor postdiagnostic MVPA, MET-h/wk					
	0 (n = 1705)	>0 to <7.5 (n = 4964)	7.5 to <15.0 (n = 2234)	15.0 to <22.5 (n = 2010)	22.5 to <30.0 (n = 1248)	≥30.0 (n = 4980)
Age at diagnosis, mean (SD), y	71.6 (9.4)	67.7 (9.5)	66.5 (8.4)	67.3 (8.9)	66.8 (8.5)	66.7 (7.1)
Sex, No. (%)						
Female	1165 (68)	3582 (72)	1375 (62)	1205 (60)	702 (56)	2202 (44)
Male	540 (32)	1382 (28)	859 (38)	805 (40)	546 (44)	2778 (56)
Race and ethnicity						
Non-Latino White, No. (%)	1649 (97)	4739 (96)	2106 (94)	1935 (96)	1187 (95)	4712 (95)
All other	56 (3)	225 (4)	128 (6)	75 (4)	61 (5)	268 (5)
Time from diagnosis to survey, mean (SD), mo	26.1 (12.5)	27.8 (14.1)	31.6 (19.2)	32.3 (18.9)	36.4 (23.4)	44.3 (25.9)
Prediagnostic BMI, mean (SD)^a^	27.6 (5.5)	26.7 (5.2)	26.4 (5.0)	26.1 (4.7)	26.2 (5.0)	26.3 (4.9)
Postdiagnostic smoking status, No. (%)						
Never	583 (34)	1855 (37)	839 (38)	725 (36)	441 (35)	1556 (31)
Former	663 (39)	2072 (42)	974 (44)	935 (46)	597 (48)	2541 (51)
Current	450 (26)	996 (20)	387 (17)	322 (16)	190 (15)	738 (15)
Missing	9 (0)	41 (1)	34 (2)	28 (1)	20 (2)	145 (3)
Postdiagnostic alcohol use, No. (%)						
User	633 (37)	1752 (35)	653 (29)	575 (29)	357 (29)	1100 (22)
Nonuser	948 (56)	2938 (59)	1473 (66)	1336 (66)	842 (68)	3771 (76)
Missing	124 (7)	274 (6)	108 (5)	99 (5)	49 (4)	109 (2)
First-course treatment, No. (%)						
Chemotherapy plus radiation	151 (9)	336 (7)	99 (4)	64 (3)	47 (4)	299 (6)
Chemotherapy only	60 (4)	253 (5.	106 (5)	62 (3)	38 (3)	258 (5)
Radiation only	72 (4)	112 (2)	58 (3)	38 (2)	23 (2)	272 (6)
No chemotherapy or radiation	300 (18)	1004 (20)	421 (19)	306 (15)	292 (23)	2139 (43)
Missing	1122 (66)	3259 (66)	1550 (69)	1540 (77)	848 (68)	2012 (40)
SEER General Summary Stage, No. (%)						
In situ	203 (12)	592 (12)	299 (13)	317 (16)	178 (14)	644 (13)
Localized	538 (32)	1659 (33)	786 (35)	708 (35)	400 (32)	1551 (31)
Regional	217 (13)	518 (10)	239 (11)	210 (10)	115 (9)	520 (10)
Local or regional^b^	114 (7)	417 (8)	158 (7)	107 (5)	75 (6)	102 (2)
Distant	177 (10)	468 (9)	149 (7)	131 (6)	67 (5)	196 (4)
Missing	456 (27)	1310 (26)	603 (27)	537 (27)	413 (33)	1967 (40)
Cancer type, No. (%)						
Bladder	365 (21)	952 (19)	520 (23)	529 (26)	338 (27)	1487 (30)
Endometrial	331 (19)	1277 (26)	561 (25)	456 (23)	251 (20)	810 (16)
Kidney	151 (8.8)	500 (10)	221 (10)	215 (11)	135 (11)	566 (11)
Lung	447 (26.2)	958 (19)	355 (16)	297 (15)	195 (16)	745 (15)
Oral	99 (6)	284 (6)	153 (7)	133 (7)	80 (6)	381 (8)
Ovarian	146 (9)	519 (10)	176 (8)	148 (8)	89 (7)	256 (5)
Rectal	166 (9.7)	474 (10)	248 (11)	232 (12)	160 (13)	735 (15)

Abbreviations: BMI, body mass index; MET-h/wk, metabolic equivalent of task hours per week; MVPA, moderate to vigorous intensity physical activity; SEER, Surveillance, Epidemiology, and End Results.

^a^ BMI calculated as weight in kilograms divided by height in meters squared.

^b^ SEER General Summary Stage could not be discerned between local or regional for certain bladder and lung cancer cases.

### Postdiagnostic Physical Activity and Cancer Mortality

**Figure 1.**
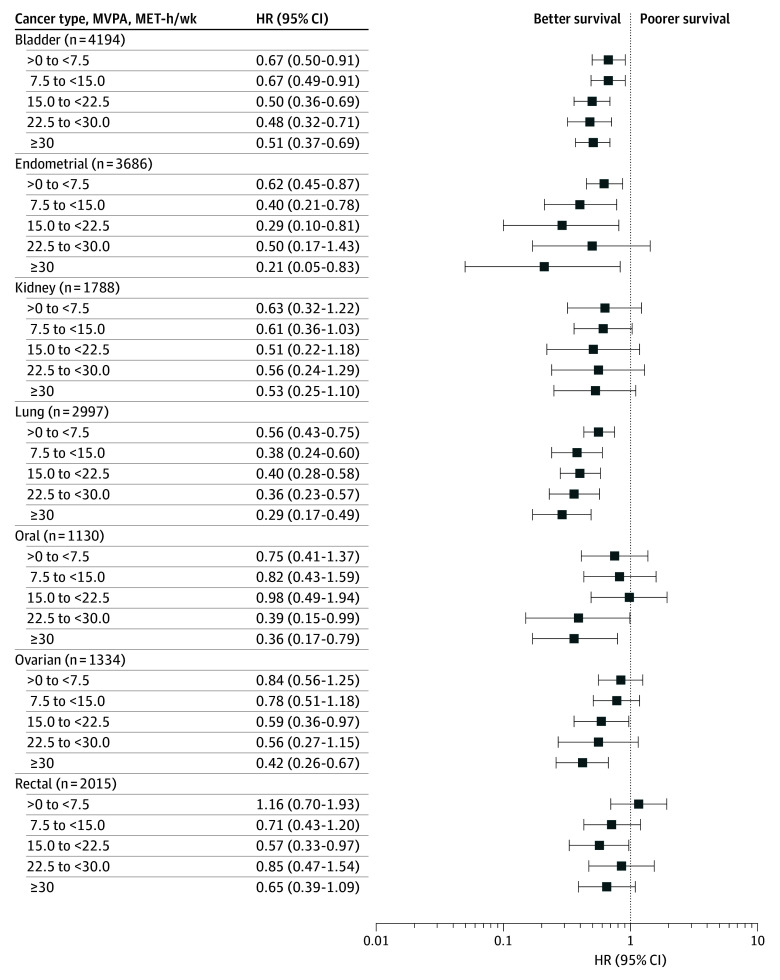
Forest Plot of Postdiagnosis Leisure-Time Moderate to Vigorous Intensity Physical Activity (MVPA) and Total Cancer Mortality Among Survivors of Bladder, Endometrial, Kidney, Lung, Oral Cavity, Ovarian, or Rectal Cancer The reference category was no MVPA. Overall *P* value for bladder cancer, *P* = .003; for endometrial cancer, *P* = .02; for kidney cancer, *P* = .53; for lung cancer, *P* < .001; for oral cancer, *P* = .06; for ovarian cancer, *P* = .007; and for rectal cancer, *P* = .30. HR indicates hazard ratio; MET-h/wk, metabolic equivalent of task hours per week.

Higher levels of postdiagnostic MVPA were associated with lower risk of cancer mortality among survivors of bladder, endometrial, lung, and ovarian cancers (Figure 1). Compared with survivors reporting no PA, any amount of PA, including an amount below recommendations (ie, >0 to <7.5 MET-h/wk), was associated with lower risk of cancer mortality among survivors of bladder (HR, 0.67 [95% CI, 0.50-0.91]), endometrial (HR, 0.62 [95% CI, 0.45-0.87]), and lung (HR, 0.56 [95% CI, 0.43-0.75]) cancer. Similarly, meeting MVPA guidelines (7.5 MET-h/wk to <15.0 MET-h/wk) was associated with further reduction in risk for endometrial (HR, 0.40 [95% CI, 0.21-0.78]) and lung (HR, 0.38 [95% CI, 0.24-0.60]) cancer survivors (Figure 1). Doubling the recommended MVPA guideline or more (eg, >15 vs 0 MET-h/wk) was associated with lower risk of cancer mortality among oral (HR, 0.39 [95% CI, 0.15-0.99] for >22.5 to 30.0 MET-h/wk) and rectal (HR, 0.57 [95% CI, 0.33-0.97] for >15.0 to 22.5 MET-h/wk) cancer survivors.

Although postdiagnostic MVPA was not associated with lower risk of cancer mortality overall for survivors of kidney, oral, or rectal cancer, the direction of each estimate was consistent with lower risk. For example, point estimates for meeting MVPA guidelines ranged from 0.61 to 0.82 for those 3 groups (eg, HR, 0.61 [95% CI, 0.36-1.03] for risk among kidney cancer survivors meeting guidelines). Results were largely similar when further adjusting for prediagnostic BMI among survivors of most cancer types (eTable 6 in Supplement 1). In an analysis exploring the influence of each cohort’s effect estimate on the overall pooled estimate, excluding 1 cohort in turn only modestly affected HRs (eTable 7 in Supplement 1).

### Prediagnostic to Postdiagnostic Changes in Physical Activity and Cancer Mortality

**Figure 2.**
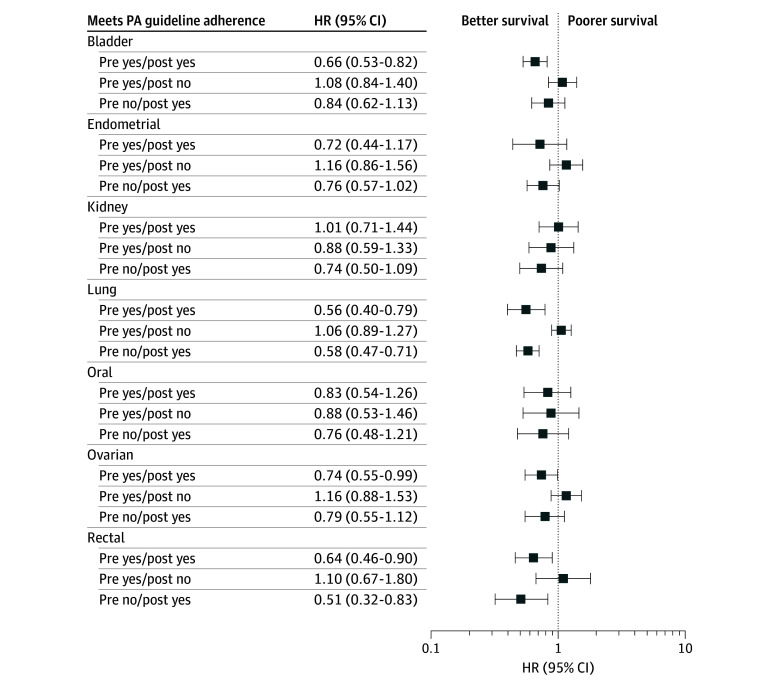
Forest Plot of Physical Activity (PA) Guideline Adherence Before (Pre) and After (Post) Diagnosis and Total Cancer Mortality Among Survivors of Bladder, Endometrial, Kidney, Lung, Oral Cavity, Ovarian, or Rectal Cancer The reference category was not meeting PA guideline adherence prediagnosis and postdiagnosis. HR indicates hazard ratio.

Given the limited clinical utility of potential findings, models using prediagnostic MVPA as the primary exposure are presented in eTable 8 and the eFigure in Supplement 1 (n = 55 709 cancer survivors). Compared with cancer survivors who did not meet the PA guidelines both before and after diagnosis, lung (HR, 0.58 [95% CI, 0.47-0.71]) and rectal (HR, 0.51 [95% CI, 0.32-0.83]) cancer survivors who met guidelines after diagnosis had lower risk of cancer mortality, even if they were inactive before the diagnosis (Figure 2). For ovarian and bladder cancer survivors, meeting guidelines postdiagnosis was associated with lower risk of cancer mortality among those who also met guidelines before diagnosis (HR, 0.66 [95% CI, 0.52-0.82] for bladder cancer; HR, 0.74 [95% CI, 0.55-0.99] for ovarian cancer). No associations were found among survivors who did not meet guidelines before diagnosis for bladder (HR, 0.84 [95% CI, 0.62-1.13) and ovarian (HR, 0.79 [95% CI, 0.55-1.12]) cancer. Although the confidence intervals included the null, point estimates suggested that endometrial cancer survivors who met guidelines after diagnosis, regardless of MVPA levels prior to diagnosis, may be at lower risk of cancer mortality compared with those who were inactive at both time points (HR, 0.76 [95% CI, 0.57-1.02] for those not meeting guidelines before diagnosis but meeting guidelines after diagnosis; HR, 0.72, [95% CI, 0.44-1.17] for those meeting both prediagnosis and postdiagnosis guidelines). There were no clear differences in risk of total cancer mortality by prediagnostic to postdiagnostic change in MVPA among survivors of kidney or oral cancer.

### Stratified and Sensitivity Analyses of Postdiagnostic Physical Activity and Cancer Mortality

Compared with main analyses, restricting to never smokers resulted in an attenuation of point estimates among endometrial cancer survivors, and there was no longer an association (Table 2). For bladder cancer survivors, restricting to never smokers strengthened point estimates, although confidence intervals widened (and included the null for those meeting and more than tripling guidelines). When excluding deaths that occurred in the first 2 years of follow-up (eg, death within 2 years of the postdiagnostic MVPA survey), HRs were attenuated, although associations remained for survivors of endometrial, lung, ovarian, and rectal cancer (Table 2). Similarly, HRs were attenuated but associations remained for survivors of kidney, lung, and rectal cancer when excluding those missing stage information and those diagnosed with distant stage disease (eTable 9 in Supplement 1) or for survivors of bladder, endometrial, lung, and ovarian cancer when accounting for competing risks (eTable 10 in Supplement 1).

**Table 2.** Stratified and Sensitivity Analyses of Postdiagnostic Physical Activity

Postdiagnostic MVPA, MET-h/wk	Hazard ratio (95% CI)	
	Excluding deaths in 2 y	Among never smokers
**Bladder cancer**		
No. of cancer deaths	769	229
0	1 [Reference]	1 [Reference]
>0 to <7.5	0.69 (0.38-1.25)	0.39 (0.17-0.88)
7.5 to <15.0	0.76 (0.41-1.42)	0.54 (0.24-1.24)
15.0 to <22.5	0.62 (0.33-1.14)	0.33 (0.14-0.80)
22.5 to <30.0	0.65 (0.32-1.34)	0.26 (0.08-0.91)
≥30.0	0.68 (0.46-1.03)	0.52 (0.21-1.29)
**Endometrial cancer**		
No. of cancer deaths	360	272
0	1 [Reference]	1 [Reference]
>0 to <7.5	0.60 (0.40-0.92)	0.80 (0.49-1.30)
7.5 to <15.0	0.56 (0.35-0.91)	0.54 (0.30-0.99)
15.0 to <22.5	0.54 (0.32-0.92)	0.73 (0.38-1.38)
22.5 to <30.0	0.82 (0.46-1.47)	0.84 (0.39-1.81)
≥30.0	0.50 (0.28-0.88)	0.52 (0.13-2.04)
**Kidney cancer**		
No. of cancer deaths	348	121
0	1 [Reference]	NA^a^
>0 to <7.5	0.57 (0.23-1.38)	NA^a^
7.5 to <15.0	0.53 (0.28-1.00)	NA^a^
15.0 to <22.5	0.53 (0.28-0.99)	NA^a^
22.5 to <30.0	0.63 (0.29-1.35)	NA^a^
≥30.0	0.54 (0.23-1.28)	NA^a^
**Lung cancer**		
No. of cancer deaths	763	114
0	1 [Reference]	NA^a^
>0 to <7.5	0.62 (0.46-0.84)	NA^a^
7.5 to <15.0	0.68 (0.47-0.99)	NA^a^
15.0 to <22.5	0.48 (0.31-0.72)	NA^a^
22.5 to <30.0	0.65 (0.41-1.04)	NA^a^
≥30.0	0.49 (0.33-0.74)	NA^a^
**Ovarian cancer**		
No. of cancer deaths	391	111
0	1 [Reference]	NA^a^
>0 to <7.5	0.61 (0.26-1.42)	NA^a^
7.5 to <15.0	0.62 (0.22-1.80)	NA^a^
15.0 to <22.5	0.55 (0.20-1.52)	NA^a^
22.5 to <30.0	0.48 (0.17-1.35)	NA^a^
≥30.0	0.35 (0.17-0.74)	NA^a^
**Rectal cancer**		
No. of cancer deaths	393	149
0	1 [Reference]	NA^a^
>0 to <7.5	0.84 (0.35-1.99)	NA^a^
7.5 to <15.0	0.52 (0.25-1.06)	NA^a^
15.0 to <22.5	0.47 (0.24-0.91)	NA^a^
22.5 to <30.0	0.67 (0.28-1.63)	NA^a^
≥30.0	0.55 (0.29-1.05)	NA^a^

Abbreviations: MET-h/wk, metabolic equivalent of task hours per week; NA, not available.

^a^ Selected stratified or sensitivity analyses are not shown because of small numbers of cancer deaths; too few cancer deaths among oral cancer survivors remained after making additional exclusions or stratifying to consider any of these analyses.

## Discussion

In this pooled analysis of 6 cohorts including 17 141 survivors of bladder, endometrial, kidney, lung, oral, ovarian, or rectal cancer, more MVPA after a diagnosis was associated with lower risk of cancer mortality among survivors of bladder, endometrial, lung, and ovarian cancer during a mean follow-up of 10.9 years. Additionally, MVPA equivalent to, double, or triple the recommended amount was associated with lower cancer mortality among oral cancer survivors. Even among participants who did not meet the MVPA guideline prior to diagnosis, meeting the guideline after diagnosis was associated with lower risk of cancer mortality for survivors of lung, and rectal cancer. These findings build on prior work by members of our team assessing associations between postdiagnostic MVPA with all-cause mortality among all cancer survivors.^32^

Findings from the current study confirm and extend with results from the small number of prior studies on postdiagnostic MVPA and cancer mortality by cancer type.^34,35,36,37^ In 1 study of 667 kidney cancer survivors, accumulating at least 7 h/wk of MVPA postdiagnosis was not associated with lower risk of cancer mortality (compared with <1 h/wk; HR, 0.59 [95% CI, 0.25-1.38]).^34^ Although our sample of kidney cancer survivors was larger, all confidence intervals similarly included the null. One study of MVPA and cancer-specific mortality among ovarian cancer survivors^35^ also reported results similar to the current study, although this is to be expected as the prior study used NHS and NHSII data, which are included in the harmonized cohort in the current study. For survivors of endometrial cancer, studies have assessed only postdiagnostic MVPA and overall survival.^36,37^ Importantly, several of these prior studies were limited in their ability to assess the association of reverse causality and residual confounding by smoking through sensitivity and stratified analyses because of a relatively low number of cancer deaths. In the current study, although we excluded deaths within the first 2 years of follow-up and restricted analyses to never smokers among bladder and endometrial cancer survivors, wide confidence intervals made it difficult to infer the impact of these 2 important biases.

Several prior cancer survival studies focused on associations with prediagnostic measures of MVPA.^23^ However, the clinical relevance of these studies may be minimal—perhaps only useful for identifying patients with cancer at the highest risk of poor prognosis—as prediagnostic behavior cannot be intervened on at the time of diagnosis.^38^ Further, survival models including prediagnostic exposures may be subject to immortal time or selection bias.^33,39^ As implications of findings around prediagnostic PA are limited, we presented associations as prediagnostic to postdiagnostic changes in PA, and presented associations with prediagnostic PA only in Supplement 1.

Lung and kidney cancer survivors who met the PA guidelines after diagnosis, but not prior to diagnosis, had a lower risk of cancer mortality compared with survivors not meeting guidelines at either time point. These findings outline an actionable and translatable public health message that may motivate cancer survivors to become as active as they are able after a diagnosis, even if they were not active before. On the other hand, meeting PA guidelines only before diagnosis (ie, not meeting guidelines after diagnosis) was not associated with a reduced likelihood of cancer mortality for survivors of any cancer type. These findings reflect results from other studies that report an inverse association between postdiagnostic MVPA and mortality but not between prediagnostic MVPA and mortality.^35,36^

### Strengths and Limitations

The primary strength of this study is the large sample of survivors of cancers less commonly studied in the context of PA and mortality, including bladder, endometrial, kidney, lung, oral, ovarian, and rectal cancer. Additionally, all included cohort studies had more than a mean decade of follow-up and had similar processes for collecting MVPA exposure, cancer outcome, and covariate data. All survivors also had cancer stage or treatment data; although treatment is an obvious estimator of mortality, these data are not always available in large cohorts and thus have historically been difficult to include in studies of MVPA and cancer mortality.

The results of this study should be interpreted with its limitations in mind. Perhaps the most substantial limitation is the likelihood for reverse causality, in which inactivity may be a marker of poor general health or impending death, resulting in spurious associations between low or no PA and cancer mortality. In the current study, follow-up time began at the postdiagnostic survey return (which occurred a mean of 2.8 years after diagnosis), but to further reduce the association with reverse causality, a sensitivity analysis excluding participants dying within 2 years of the postdiagnostic MVPA measure was examined. Results from this sensitivity analysis were indeed attenuated compared with the main results, although associations remained for survivors of most cancers besides bladder cancer. While the lag from diagnosis to postdiagnostic measures may reduce the impact of reverse causality, it may also be associated with selection bias, as patients with cancer needed to be healthy enough to complete a postdiagnostic survey. Stratified analyses also suggested that results of the main analysis may have been associated with residual confounding by smoking. Importantly, the number of cancer deaths among never smokers was too small to perform this analysis among survivors of 2 types of cancer that are highly associated with smoking: lung and oral cancer. In addition, interpretation of the current study may be limited by the reliance on a single time point of self-reported PA data, which is susceptible to recall and social desirability bias. However, validation studies of the MVPA measures used in these cohorts have shown acceptable agreement with doubly labeled water and accelerometry.^40,41,42,43,44,45,46^ Further, there is evidence to suggest that use of self-reported measures, compared with device-based measures, may result in an attenuation toward the null for certain health outcomes.^47^

## Conclusions

Findings from this pooled analysis of 6 cohort studies suggest that any amount of postdiagnostic MVPA was associated with lower risk of cancer mortality among survivors of less commonly studied cancers, including bladder, endometrial, lung, and ovarian cancers. A higher amount of postdiagnostic MVPA consistent with doubling or tripling MVPA guidelines may also be associated with lower risk of cancer mortality among survivors of oral and rectal cancer. Further research is needed to pinpoint the optimal dose of MVPA for survival among people with a history of cancer. Furthermore, there were no clear indications that postdiagnostic MVPA may be more beneficial for survivors of certain cancers over others, in part due to wide and overlapping confidence intervals. Additional research is warranted to clarify whether associations may vary by specific cancer site. Findings suggest the importance of promoting PA for longevity and overall health among people living with and beyond cancer.

## Notes

Supplement 1. **eTable 1.** Cohort and physical activity measure information **eTable 2.** International Classification of Diseases for Oncology, 3rd Ed. Codes by cancer site **eTable 3.** Cancer case numbers for models of prediagnostic physical activity by cohort **eTable 4.** Cancer case numbers for models of postdiagnostic physical activity by cohort **eTable 5.** Results from tests of the proportional hazards assumption **eTable 6.** Summary hazard ratios (HR) and 95% confidence intervals of cancer mortality for postdiagnostic moderate-to-vigorous intensity aerobic physical activity (MVPA) by cancer type, adjusting for body mass index **eTable 7.** Impact of omitting each cohort in turn from the analysis: summary hazard ratios (HR) and 95% confidence intervals of cancer mortality for postdiagnostic moderate-to-vigorous intensity aerobic physical activity (MVPA) **eTable 8.** Cancer survivor characteristics by prediagnostic leisure-time physical activity **eFigure.** Summary hazard ratios (HR) and 95% confidence intervals of cancer mortality for prediagnostic moderate-to-vigorous intensity aerobic physical activity (MVPA) by cancer type **eTable 9.** Summary hazard ratios (HR) and 95% confidence intervals of cancer mortality for postdiagnostic moderate-to-vigorous intensity aerobic physical activity (MVPA) among participants diagnosed with in situ, local, or regional stage cancer **eTable 10.** Summary hazard ratios (HR) and 95% confidence intervals of cancer mortality for postdiagnostic moderate-to-vigorous intensity aerobic physical activity (MVPA), Fine and Gray models of competing risk

Supplement 2.
Data Sharing Statement
