# Supplementary material for: Leisure-Time Physical Activity and Cancer Mortality Among Cancer Survivors
Source: JAMA Netw Open. 2026 Feb 17;9(2):e2556971. doi: 10.1001/jamanetworkopen.2025.56971 (PMC12914486; doi:10.1001/jamanetworkopen.2025.56971)
Supplement: Supplement 1. — eTable 1. Cohort and physical activity measure information eTable 2. International Classification of Diseases for Oncology, 3rd Ed. Codes by cancer site eTable 3. Cancer case numbers for models of prediagnostic physical activity by cohort eTable 4. Cancer case numbers for models of postdiagnostic physical activity by cohort eTable 5. Results from tests of the proportional hazards assumption eTable 6. Summary hazard ratios (HR) and 95% confidence intervals of cancer mortality for postdiagnostic moderate-to-vigorous intensity aerobic physical activity (MVPA) by cancer type, adjusting for body mass index eTable 7. Impact of omitting each cohort in turn from the analysis: summary hazard ratios (HR) and 95% confidence intervals of cancer mortality for postdiagnostic moderate-to-vigorous intensity aerobic physical activity (MVPA) eTable 8. Cancer survivor characteristics by prediagnostic leisure-time physical activity eFigure. Summary hazard ratios (HR) and 95% confidence intervals of cancer mortality for prediagnostic moderate-to-vigorous intensity aerobic physical activity (MVPA) by cancer type eTable 9. Summary hazard ratios (HR) and 95% confidence intervals of cancer mortality for postdiagnostic moderate-to-vigorous intensity aerobic physical activity (MVPA) among participants diagnosed with in situ, local, or regional stage cancer eTable 10. Summary hazard ratios (HR) and 95% confidence intervals of cancer mortality for postdiagnostic moderate-to-vigorous intensity aerobic physical activity (MVPA), Fine and Gray models of competing risk [file jamanetwopen-e2556971-s001.pdf]

Supplementary Online Content

Rees-Punia E, Teras LR, Newton CC, et al. Leisure-time physical activity and cancer mortality among cancer survivors. *JAMA Netw Open*. 2026;9(2):e2556971.
doi:10.1001/jamanetworkopen.2025.56971

eTable 1. Cohort and physical activity measure information

eTable 2. International Classification of Diseases for Oncology, 3rd Ed. Codes by cancer site

eTable 3. Cancer case numbers for models of prediagnostic physical activity by cohort

eTable 4. Cancer case numbers for models of postdiagnostic physical activity by cohort

eTable 5. Results from tests of the proportional hazards assumption

eTable 6. Summary hazard ratios (HR) and 95% confidence intervals of cancer mortality for postdiagnostic moderate-to-vigorous intensity aerobic physical activity (MVPA) by cancer type, adjusting for body mass index

eTable 7. Impact of omitting each cohort in turn from the analysis: summary hazard ratios (HR) and 95% confidence intervals of cancer mortality for postdiagnostic moderate-to-vigorous intensity aerobic physical activity (MVPA)

eTable 8. Cancer survivor characteristics by prediagnostic leisure-time physical activity

eFigure. Summary hazard ratios (HR) and 95% confidence intervals of cancer mortality for prediagnostic moderate-to-vigorous intensity aerobic physical activity (MVPA) by cancer type

eTable 9. Summary hazard ratios (HR) and 95% confidence intervals of cancer mortality for postdiagnostic moderate-to-vigorous intensity aerobic physical activity (MVPA) among participants diagnosed with in situ, local, or regional stage cancer

eTable 10. Summary hazard ratios (HR) and 95% confidence intervals of cancer mortality for postdiagnostic moderate-to-vigorous intensity aerobic physical activity (MVPA), Fine and Gray models of competing risk

eReferences.

This supplementary material has been provided by the authors to give readers additional information about their work.

© 2026 Rees-Punia E et al. *JAMA Network Open*.

eTable 1. Cohort and physical activity measure information

	Cancer Prevention Study-II (CPS-II)	Health Professionals Follow-up Study (HPFS)	NIH-AARP Diet and Health Study (NIH-AARP)	Nurses' Health Study (NHS)	Nurses' Health Study II (NHSII)	Women's Health Study (WHS)
**Baseline year(s)**	1992-1993	1986	1995-1997	1976	1989	1992
**Baseline participants**	154,425	51,529	507,826	121,700	116,430	39,414
**Baseline paper (case ascertainment details)**	Calle 2002(1)	Giovannucci 1993(2)	Schatzkin 2001(3)	Belanger 1978(4)	Belanger 1978(4); Garland, 1999(5)	Rexrode 2000(6)
**Physical activity measure validation paper**	Bonnefoy 2001(7)	Chasan-Taber 1996(8); Pernar 2022(9)	Milton 2013(10)	Wolf 1994(11); Al-Shaar 2022(12)	Wolf 1994(11); Al-Shaar 2022(12)	Bonnefoy 2001(7); Shiroma 2016(13)
**Frequency of activity assessment**	Every 2 years starting in 1997 (except 2003 and 2007)	Every 2 years starting in 1990	Baseline, 2004-2006	Every 2 years	Every 2 years	Every 2-4 years
**PA question details**	"During the past year, what was your average total time per week spent at each of the following activities?", including 7 moderate-vigorous intensity PA items and 9 response options in time/week.	"During the past year, what was your average total time per week spent at each activity?", including 7 moderate-vigorous intensity PA items and 8 response options in time/week.	"During the past year, what was your average time per week spent at each of the following recreational activities?", including 8 moderate-vigorous intensity PA items and 10 response items in time/week.	"During the past year, what was your average time per week spent at each of the following recreational activities?", including 8 moderate-vigorous intensity PA items and 10 response items in time/week.	"During the past year, what was your average time per week spent at each of the following recreational activities?", including 8 moderate-vigorous intensity PA items and 10 response items in time/week.	"During the past year, what was your approximate time per week spent at each of the following recreational activities?", including 7 moderate-vigorous intensity PA items and 8 response options in time/week.

© 2026 Rees-Punia E et al. *JAMA Network Open*.

eTable 2. International Classification of Diseases for Oncology, 3rd Ed. Codes by cancer site

**Cancer Site**	**ICD-O-3 Codes**
**Bladder**	C670-C679
**Endometrial**	C540-C549 and C559
**Kidney**	C649 and C659
**Lung**	C340-C349
**Oral cavity (lip, tongue, gum, floor of mouth, palate, salivary gland, tonsil, nasopharynx, hypopharynx, other oral cavity)**	C000-C009, C010, C020-C029, C030-C039, C040-C049, C050-C059, C060-C069, C070, C080-C089, C090-C099, C100-C109, C110-C119, C120, C130-C139, C140, C148
**Ovary**	C569
**Rectum, anus, anal cavity**	C199, C209, C211-C218

eTable 3. Cancer case numbers for models of prediagnostic physical activity by cohort

**Cancer Type**	**AARP**	**CPS-II**	**HPFS**	**NHS**	**NHSII**	**WHS**	**Total**
Bladder	6577	2132	1055	530	239	NA[2]	10533
Endometrial	2200	1170	NA[1]	1473	933	586	6362
Kidney	3110	852	369	389	210	136	5066
Lung	14463	3693	1107	2183	335	628	22409
Oral	2052	497	215	213	168	NA[2]	3145
Ovary	1124	613	NA[1]	753	519	272	3281
Rectal	3013	937	248	373	189	153	4913

[1]Sex-specific cancer type not included in this cohort.
[2]Less than 25 cancer deaths among survivors of this cancer type with complete treatment and/or stage data in this cohort.

© 2026 Rees-Punia E et al. *JAMA Network Open*.

eTable 4. Cancer case numbers for models of postdiagnostic physical activity by cohort

Cancer Type	AARP	CPS-II	HPFS	NHS	NHSII	WHS	Total
Bladder	1526	1489	692	385	NA[2]	99	4191
Endometrial	651	841	NA[1]	1144	537	513	3686
Kidney	580	506	231	249	125	97	1788
Lung	846	949	211	638	91	262	2997
Oral	544	317	127	142	NA[2]	NA[2]	1130
Ovary	173	304	NA[1]	411	272	174	1334
Rectal	891	704	176	244	NA[2]	NA[2]	2015

[1]Sex-specific cancer type not included in this cohort.
[2]Less than 25 cancer deaths among survivors of this cancer type with complete treatment and/or stage data in this cohort.

eTable 5. Results from tests of the proportional hazards assumption

Cancer Type	AARP	CPS-II	HPFS	NHS	NHSII	WHS
Bladder	0.07	0.05	0.07	0.45	NA[3]	0.96
Endometrial	0.40	0.39	NA[2]	0.05	0.74	0.52
Kidney	0.62	0.06	0.81	0.66	0.49	0.27
Lung	**0.001**	**0.004**	0.16	0.07	0.43	0.07
Oral	**0.04**	0.60	0.84	0.15	NA[3]	NA[3]
Ovary	0.80	0.68	NA[2]	0.13	0.05	0.20
Rectal	0.20	0.08	0.93	0.71	NA[3]	NA[3]

[1] Proportional hazard assumptions of a constant exposure effect over time were explored by testing the significance of interaction terms between follow-up time and exposure.
[2]Sex-specific cancer type not included in this cohort.
[3]Less than 25 cancer deaths among survivors of this cancer type with complete treatment and/or stage data in this cohort.

© 2026 Rees-Punia E et al. *JAMA Network Open*.

eTable 6. Summary hazard ratios (HR) and 95% confidence intervals of cancer mortality for postdiagnostic moderate-to-vigorous intensity aerobic physical activity (MVPA) by cancer type, adjusting for body mass index

	POSTDIAGNOSTIC MVPA (MET-hr./wk.)					
**Cancer type**	**0**	**>0-<7.5**	**7.5-<15**	**15-<22.5**	**22.5-<30**	**≥30**
Bladder	Ref.	**0.70 (0.52-0.94)**	**0.70 (0.51-0.95)**	**0.52 (0.37-0.72)**	**0.52 (0.35-0.76)**	**0.53 (0.39-0.73)**
Endometrial	Ref.	**0.65 (0.47-0.91)**	**0.36 (0.16-0.82)**	**0.28 (0.10-0.83)**	0.51 (0.20-1.31)	**0.23 (0.06-0.85)**
Kidney	Ref.	0.69 (0.36-1.33)	0.63 (0.36-1.09)	0.65 (0.34-1.23)	0.68 (0.30-1.56)	0.56 (0.27-1.16)
Lung	Ref.	**0.52 (0.37-0.72)**	**0.35 (0.21-0.58)**	**0.35 (0.22-0.53)**	**0.32 (0.18-0.54)**	**0.26 (0.15-0.47)**
Oral	Ref.	0.73 (0.33-1.65)	0.80 (0.41-1.56)	1.03 (0.51-2.06)	0.40 (0.16-1.04)	0.40 (0.15-1.11)
Ovarian	Ref.	0.87 (0.60-1.28)	0.82 (0.56-1.19)	**0.65 (0.43-0.97)**	0.61 (0.29-1.25)	**0.44 (0.27-0.73)**
Rectal	Ref.	1.15 (0.75-1.77)	0.67 (0.40-1.13)	**0.54 (0.32-0.93)**	0.72 (0.39-1.32)	0.66 (0.39-1.11)

MVPA: moderate-to-vigorous intensity physical activity. MET-hr./wk.: Metabolic equivalent of task- hours per week.

eTable 7. Impact of omitting each cohort in turn from the analysis: summary hazard ratios (HR) and 95% confidence intervals of cancer mortality for postdiagnostic moderate-to-vigorous intensity aerobic physical activity (MVPA)

	Overall HR (7.5 - < 15 MET-hr./wk. vs. no MVPA) when omitting cohort						
**Cancer**	**Overall**	**AARP**	**CPS-II**	**HPFS**	**NHS**	**NHSII**	**WHS**
Bladder	0.67 (0.49-0.91)	0.67 (0.49-0.92)	0.51 (0.31-0.83)	0.73 (0.51-1.02)	0.68 (0.49-0.94)	NA[2]	0.67 (0.49-0.91)
Endometrial	0.40 (0.21-0.78)	0.51 (0.34-0.76)	0.28 (0.09-0.85)	NA[1]	0.28 (0.09-0.88)	0.48 (0.34-0.68)	0.44 (0.22-0.90)
Kidney	0.61 (0.36-1.03)	0.63 (0.36-1.11)	0.56 (0.26-1.23)	0.69 (0.38-1.23)	0.53 (0.30-0.95)	NA[2]	0.61 (0.35-1.03)
Lung	0.38 (0.24-0.60)	0.40 (0.23-0.67)	0.33 (0.23-0.47)	0.42 (0.26-0.68)	0.34 (0.18-0.66)	0.40 (0.25-0.63)	0.39 (0.23-0.66)
Oral cavity	0.82 (0.43-1.59)	0.83 (0.42-1.64)	0.48 (0.13-1.72)	0.82 (0.42-1.60)	0.98 (0.47-2.04)	NA[2]	NA[2]
Ovary	0.78 (0.51-1.18)	0.77 (0.48-1.25)	0.72 (0.37-1.43)	NA[1]	0.66 (0.37-1.19)	0.91 (0.62-1.32)	0.72 (0.44-1.17)
Rectal	0.71 (0.43-1.20)	0.75 (0.43-1.29)	0.48 (0.21-1.12)	0.75 (0.43-1.30)	0.78 (0.45-1.36)	NA[2]	NA[2]

[1]Sex-specific cancer type not captured in this cohort.
[2]Less than 25 cancer deaths among survivors of this cancer type with complete treatment and/or stage data in this cohort.

eTable 8. Cancer survivor characteristics by prediagnostic leisure-time physical activity

	**Prediagnostic MVPA**					
	**0 MET-hr./wk.**	**>0 - < 7.5 MET-hr./wk.**	**7.5 – <15 MET-hr./wk.**	**15 - <22.5 MET-hr./wk.**	**22.5 - <30 MET-hr./wk.**	**≥30 MET-hr./wk.**
	n = 4274	n = 15495	n = 8508	n = 9268	n = 10148	n = 8016
	**Mean (SD)**					
**Age at Diagnosis (years)**	69.9 (7.7)	69.4 (8.4)	69.8 (8.0)	70.1 (7.5)	70.9 (7.1)	69.9 (8.2)
**Time from survey to diagnosis (months)**	115.9 (79.3)	133.4 (85.2)	120.0 (76.0)	110.9 (69.5)	105.2 (64.5)	115.8 (73.0)
**Prediagnostic Body Mass Index (kg/m^2)**	27.3 (5.7)	27.1 (5.4)	26.8 (5.1)	26.8 (4.8)	26.4 (4.4)	25.8 (4.4)
	**No. (%)**					
**Women**	2286 (53.5%)	9341 (60.3%)	4229 (49.7%)	4046 (43.7%)	3851 (37.9%)	3173 (39.6%)
**White/non-Latino**	3972 (92.9%)	14647 (94.5%)	8068 (94.8%)	8763 (94.6%)	9553 (94.1%)	7528 (93.9%)
**Prediagnostic smoking status**						
Never Smokers	855 (20.0%)	4168 (26.9%)	2157 (25.4%)	2087 (22.5%)	2241 (22.1%)	1925 (24.0%)
Former Smokers	1769 (41.4%)	6505 (42.0%)	4002 (47.0%)	4586 (49.5%)	5518 (54.4%)	4343 (54.2%)
Current Smokers	1564 (36.6%)	4569 (29.5%)	2185 (25.7%)	2327 (25.1%)	2075 (20.4%)	1484 (18.5%)
Missing	86 (2.0%)	253 (1.6%)	164 (1.9%)	268 (2.9%)	314 (3.1%)	264 (3.3%)
**Prediagnostic alcohol use**						
User	1571 (36.8%)	4827 (31.1%)	2243 (26.4%)	2136 (23.0%)	2348 (23.1%)	2034 (25.4%)
Non-user	2459 (57.5%)	9941 (64.2%)	6012 (70.7%)	7000 (75.5%)	7643 (75.3%)	5849 (73.0%)
Missing	244 (5.7%)	727 (4.7%)	253 (3.0%)	132 (1.4%)	157 (1.5%)	133 (1.7%)
**Cancer Type**						
Bladder	648 (15.2%)	2528 (16.3%)	1551 (18.2%)	1867 (20.1%)	2187 (21.6%)	1752 (21.9%)
Endometrial	461 (10.8%)	2405 (15.5%)	1056 (12.4%)	898 (9.7%)	814 (8.0%)	728 (9.1%)
Kidney	315 (7.4%)	1351 (8.7%)	760 (8.9%)	889 (9.6%)	1017 (10.0%)	734 (9.2%)
Lung	2056 (48.1%)	6065 (39.1%)	3348 (39.4%)	3777 (40.8%)	4107 (40.5%)	3056 (38.1%)
Oral	223 (5.2%)	775 (5.0%)	512 (6.0%)	536 (5.8%)	607 (6.0%)	492 (6.1%)
Ovarian	211 (4.9%)	1134 (7.3%)	539 (6.3%)	470 (5.1%)	467 (4.6%)	460 (5.7%)
Rectal	360 (8.4%)	1237 (8.0%)	742 (8.7%)	831 (9.0%)	949 (9.4%)	794 (9.9%)

MVPA: moderate-to-vigorous intensity physical activity. MET-hr./wk.: Metabolic equivalent of task- hours per week.

© 2026 Rees-Punia E et al. *JAMA Network Open*.

eFigure. Summary hazard ratios (HR) and 95% confidence intervals of cancer mortality for prediagnostic moderate-to-vigorous intensity aerobic physical activity (MVPA) by cancer type. Shading indicates the recommended amount of MVPA.

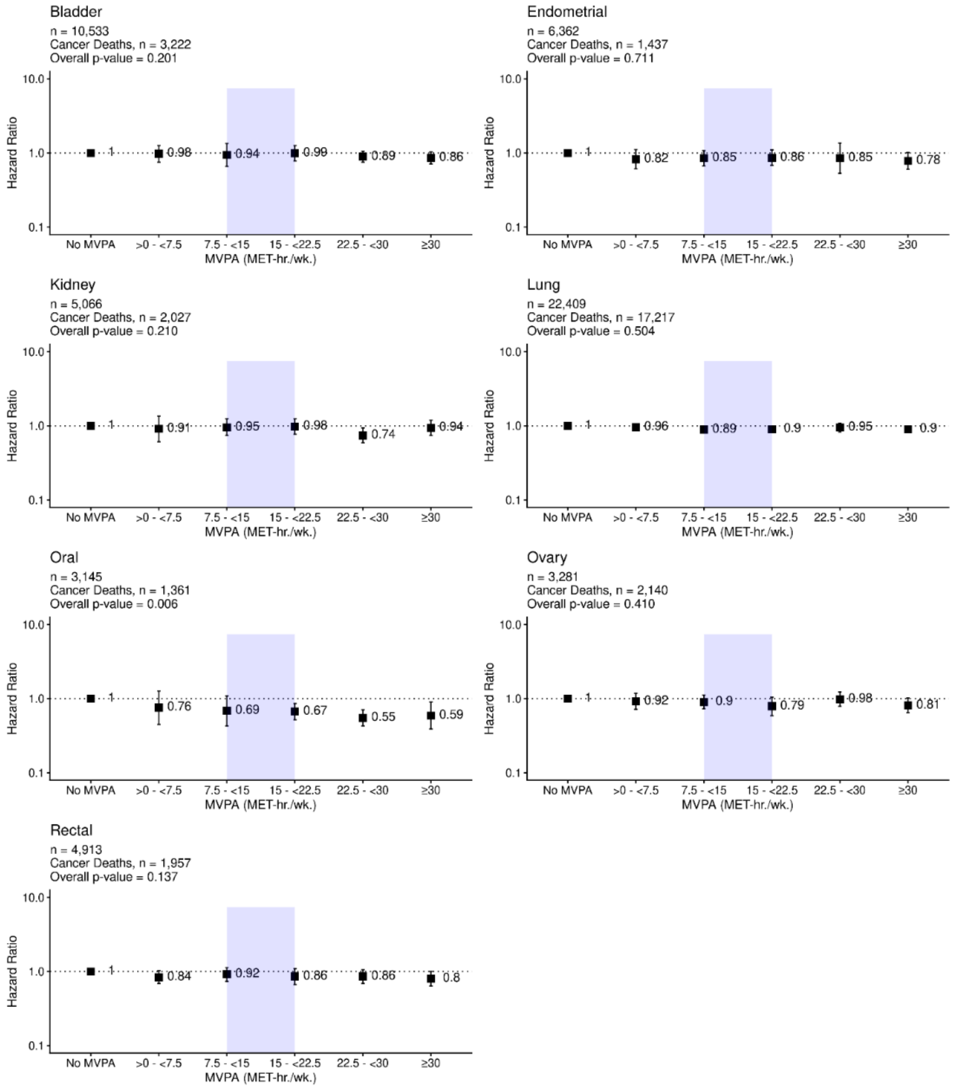

© 2026 Rees-Punia E et al. *JAMA Network Open*.

eTable 9. Summary hazard ratios (HR) and 95% confidence intervals of cancer mortality for postdiagnostic moderate-to-vigorous intensity aerobic physical activity (MVPA) among participants diagnosed with in situ, local, or regional stage cancer

	POSTDIAGNOSTIC MVPA (MET-hr./wk.)		
**Cancer type**	**<7.5**	**≥7.5 - <15**	**≥15**
Bladder	Ref.	0.94 (0.60-1.47)	0.68 (0.46-1.02)
Endometrial	Ref.	0.77 (0.52-1.13)	0.91 (0.63-1.32)
Kidney	Ref.	0.51 (0.20-1.28)	**0.46 (0.24-0.87)**
Lung	Ref.	0.96 (0.71-1.30)	**0.59 (0.45-0.76)**
Ovarian	Ref.	2.51 (0.70-9.06)	1.06 (0.45-2.45)
Rectal	Ref.	0.77 (0.49-1.20)	**0.66 (0.46-0.93)**

Too few cancer deaths among oral cancer survivors remained after making additional exclusions to consider above analysis. MVPA: moderate-to-vigorous intensity physical activity. MET-hr./wk.: Metabolic equivalent of task- hours per week.

eTable 10. Summary hazard ratios (HR) and 95% confidence intervals of cancer mortality for postdiagnostic moderate-to-vigorous intensity aerobic physical activity (MVPA), Fine and Gray models of competing risk

	POSTDIAGNOSTIC MVPA (MET-hr./wk.)					
**Cancer type**	**0**	**>0-<7.5**	**7.5-<15**	**15-<22.5**	**22.5-<30**	**≥30**
Bladder	Ref.	0.80 (0.62-1.03)	0.84 (0.64-1.11)	**0.66 (0.50-0.89)**	**0.68 (0.49-0.93)**	**0.74 (0.57-0.95)**
Endometrial	Ref.	0.73 (0.54-1.00)	**0.67 (0.47-0.97)**	**0.56 (0.37-0.84)**	0.78 (0.50-1.21)	**0.65 (0.46-0.94)**
Kidney	Ref.	0.73 (0.51-1.03)	0.72 (0.47-1.08)	0.74 (0.49-1.11)	0.77 (0.48-1.22)	**0.67 (0.46-0.97)**
Lung	Ref.	**0.70 (0.59-0.83)**	**0.59 (0.48-0.74)**	**0.57 (0.45-0.72)**	**0.59 (0.48-0.74)**	**0.49 (0.41-0.59)**
Oral	Ref.	0.95 (0.58-1.54)	0.93 (0.55-1.58)	0.96 (0.57-1.60)	0.80 (0.44-1.46)	0.80 (0.50-1.28)
Ovarian	Ref.	0.86 (0.67-1.11)	0.80 (0.59-1.08)	**0.67 (0.48-0.94)**	0.68 (0.47-1.00)	**0.59 (0.43-0.80)**
Rectal	Ref.	1.11 (0.79-1.56)	0.89 (0.61-1.32)	0.83 (0.56-1.23)	0.78 (0.49-1.23)	0.85 (0.60-1.22)

MVPA: moderate-to-vigorous intensity physical activity. MET-hr./wk.: Metabolic equivalent of task- hours per week.

© 2026 Rees-Punia E et al. *JAMA Network Open*.

eReferences.

1. Calle EE, Rodriguez C, Jacobs EJ, et al.: The American Cancer Society Cancer Prevention Study II Nutrition Cohort: rationale, study design, and baseline characteristics. *Cancer.* 2002, *94:*500-511.
2. Giovannucci E, Ascherio A, Rimm EB, et al.: A prospective cohort study of vasectomy and prostate cancer in US men. *JAMA.* 1993, *269:*873-877.
3. Schatzkin A, Subar AF, Thompson FE, et al.: Design and serendipity in establishing a large cohort with wide dietary intake distributions : the National Institutes of Health-American Association of Retired Persons Diet and Health Study. *Am J Epidemiol.* 2001, *154:*1119-1125.
4. Belanger CF, Hennekens CH, Rosner B, Speizer FE: The nurses' health study. *Am J Nurs.* 1978, *78:*1039-1040.
5. Garland M, Hunter DJ, Colditz GA, et al.: Alcohol consumption in relation to breast cancer risk in a cohort of United States women 25-42 years of age. *Cancer Epidemiol Biomarkers Prev.* 1999, *8:*1017-1021.
6. Rexrode KM, Lee IM, Cook NR, Hennekens CH, Buring JE: Baseline characteristics of participants in the Women's Health Study. *J Womens Health Gend Based Med.* 2000, *9:*19-27.
7. Bonnefoy M, Normand S, Pachiaudi C, et al.: Simultaneous validation of ten physical activity questionnaires in older men: a doubly labeled water study. *J Am Geriatr Soc.* 2001, *49:*28-35.
8. Chasan-Taber S, Rimm EB, Stampfer MJ, et al.: Reproducibility and Validity of a Self-Administered Physical Activity Questionnaire for Male Health Professionals. *Epidemiology.* 1996, *7:*81-86.
9. Pernar CH, Chomistek AK, Barnett JB, et al.: Validity and Relative Validity of Alternative Methods of Assessing Physical Activity in Epidemiologic Studies: Findings From the Men's Lifestyle Validation Study. *Am J Epidemiol.* 2022, *191:*1307-1322.
10. Milton K, Clemes S, Bull F: Can a single question provide an accurate measure of physical activity? *Br J Sports Med.* 2013, *47:*44-48.
11. Wolf A, Hunter D, Colditz G, et al.: Reproducibility and Validity of a Self-Administered Physical Activity Questionnaire. *International Journal of Epidemiology.* 1994, *23:*991-999.
12. Al-Shaar L, Pernar CH, Chomistek AK, et al.: Reproducibility, Validity, and Relative Validity of Self-Report Methods for Assessing Physical Activity in Epidemiologic Studies: Findings From the Women's Lifestyle Validation Study. *Am J Epidemiol.* 2022, *191:*696-710.
13. Shiroma EJ, Cook NR, Manson JE, et al.: Comparison of Self-Reported and Accelerometer-Assessed Physical Activity in Older Women. *PLOS ONE.* 2016, *10:*e0145950.

© 2026 Rees-Punia E et al. *JAMA Network Open*.
