# Supplementary material for: Leisure-Time Physical Activity and Cancer Mortality Among Cancer Survivors
Source: JAMA Netw Open. 2026 Feb 17;9(2):e2556971. doi: 10.1001/jamanetworkopen.2025.56971 (PMC12914486; doi:10.1001/jamanetworkopen.2025.56971)
Supplement: Supplement 2. — Data Sharing Statement [file jamanetwopen-e2556971-s002.pdf]

Data Sharing Statement

Rees-Punia. Leisure-Time Physical Activity and Cancer Mortality Among Cancer Survivors. *JAMA Netw Open*. Published February 17, 2026. doi:10.1001/jamanetworkopen.2025.56971

Data
Data available: No

Additional Information
Explanation for why data not available: Data for each included cohort may be available upon reasonable request.
